# Clinical significance of dual-energy dual-layer CT parameters in differentiating small-sized gastrointestinal stromal tumors from leiomyomas

**DOI:** 10.1007/s11604-023-01473-4

**Published:** 2023-07-19

**Authors:** Daisuke Tsurumaru, Yusuke Nishimuta, Satohiro Kai, Eiji Oki, Yosuke Minoda, Kousei Ishigami

**Affiliations:** 1 Department of Clinical Radiology, Graduate School of Medical Sciences, Kyushu University, 3-1-1 Maidashi, Higashi-Ku, Fukuoka, Japan; 2 Department of Surgery and Sciences, Graduate School of Medical Sciences, Kyushu University, 3-1-1 Maidashi, Higashi-Ku, Fukuoka, Japan; 3 Department of Medicine and Bioregulatory Science, Graduate School of Medical Sciences, Kyushu University, 3-1-1 Maidashi, Higashi-Ku, Fukuoka, Japan

**Keywords:** Gastrointestinal stromal tumors, Leiomyomas, Dual-energy CT, Iodine density

## Abstract

**Purpose:**

Small gastrointestinal stromal tumors (GISTs) can generally have nonspecific CT findings similar to those with benign submucosal tumors of the stomach. The purpose of this study was to explore the potential dual-layer dual-energy CT (dlDECT) parameters to differentiate small-sized (≤ 4 cm) GISTs from leiomyomas of the stomach.

**Materials and methods:**

This retrospective study included 26 SMTs ≤ 4 cm in diameter with pathological confirmation of either GIST (*n* = 17) or leiomyoma (*n* = 9) from May 2018 to January 2022. All patients received contrast-enhanced CT. The normalized iodine concentration (NIC) and spectral slope (*λ*_HU_) were compared between GIST and leiomyoma. Receiver-operating characteristic (ROC) curves were plotted and the areas under the curve (AUCs) were calculated to estimate the diagnostic performance of these markers for differentiating GISTs from leiomyomas.

**Results:**

NIC was significantly higher in GIST than in leiomyoma in the portal (*P* = 0.0019) and delayed phases (*P* = 0.0011). *λ*_HU_ was significantly higher in GIST than in leiomyoma in the portal (*P* = 0.0006) and delayed phases (*P* = 0.0009). AUC of the ROC curves using NIC to differentiate between GIST and leiomyoma were 0.875 and 0.895 in the portal and delayed phase; using *λ*_HU_, they were 0.918 and 0.902 in the portal and delayed phase.

**Conclusion:**

dlDECT parameters including NIC and *λ*_HU_ show promise as indicators for differentiating small-sized GISTs from leiomyomas.

## Introduction

Gastrointestinal stromal tumors (GISTs) are mesenchymal malignancies of the gastrointestinal tract manifesting as submucosal tumors (SMT), most frequently in the stomach. Because of the elusive nature of submucosal tumors covered with normal gastric mucosa, endoscopic biopsy is less useful and more invasive procedures, such as endoscopic ultrasonography-guided fine needle aspiration biopsy, are used to confirm pathological diagnosis before treatment. Computed tomography (CT) is indicated before or after confirmation of diagnosis as a non-invasive test to inspect local or systemic involvement. GISTs typically appear as a gastric mass with a larger size, exophytic growth, heterogeneous enhancement, necrotic areas, or cyst formation [1–3]. Benign SMTs, such as leiomyomas or schwannomas, typically show homogeneous enhancement with no intratumoral necrosis, which are different from GISTs on contrast-enhanced CT [3–7]. However, small GISTs (less than 4 or 5 cm in diameter) can generally have nonspecific CT findings [4, 6, 8, 9]. It would be clinically helpful to identify CT parameters that could differentiate small-sized GIST lesions from benign SMTs.

In recent years, detector-based dual-layer dual-energy CT (dlDECT), made possible by relatively new CT equipment, has been widely accepted as a superior diagnostic tool. It can provide specific imaging parameters, including iodine density, monoenergetic imaging, and z-effective values, which enables the characterization of tissue decomposition. Several reports have illustrated dlDECT features that can characterize GISTs or differentiate them from other tumor types [10, 11].

The purpose of this study was to explore the potential of dlDECT parameters to differentiate small-sized (≤ 4 cm) GISTs and leiomyomas of the stomach.

## Materials and methods

### Study population

This was a retrospective, single-center, observational study performed at our institution from May 2018 to January 2022. The institutional review board of our hospital approved this study and waived informed consent for all patients. The study group consisted of 35 consecutive patients who had already been confirmed to have SMTs of the stomach after gastroscopy at other clinics and who visited our institution to obtain a more specific diagnosis. The inclusion criteria consisted of patients who were evaluated by contrast-enhanced CT using the dlDECT system, whose tumors had appeared on conventional transverse-view CT as a mass ≤ 4 cm in diameter [4], and a final diagnosis of GIST or other mesenchymal tumors. Ultimately, 26 patients (17 GIST and 9 leiomyoma) were enrolled in this study. All GIST cases were diagnosed by endoscopic ultrasound-guided fine needle aspiration biopsy (EUS-FNAB) or mucosal cutting biopsy and underwent surgical resection. Pathological subtypes included 15 spindle-cell and 2 epithelioid types, rated as low risk (*n* = 3) or very low risk (*n* = 14) by the Fletcher classification. All cases of leiomyoma were diagnosed by EUS-FNAB or mucosal cutting biopsy except one case diagnosed by tumor resection. There were no significant differences in patient age, gender, tumor location, and tumor diameter between the GIST and leiomyoma cases. The patients’ clinicopathological characteristics are presented in Table 1.

**Table 1:** Demographic characteristics of gastric GIST and leiomyoma

	GIST (*n* = 17)	Leiomyoma (*n* = 9)	*P*
Age			
Median, year (range)	63 (40–81)	59 (27–72)	0.787
Gender			
Male	11	5	0.648
Female	6	4	
Location			
Cardia	0	3	0.134
Fundus	4	0	
Body	13	6	
Diameter			
Median, mm (range)	15 (8–25)	14 (8–23)	0.416
Diagnostic procedure			
Biopsy	15	9	0.766
Surgery	2	0	
Treatment			
Conservative	0	8	< 0.001
Surgery	17	1	

### dlDECT images

All patients underwent dlDECT scanning (iQon Spectral CT; Philips Healthcare, Best, The Netherlands). Before CT, they drank 500 ml of water to distend the stomach. The scanning was performed before and after the intravenous administration of 600 mgI/kg of iodinated contrast material (Iopamiron 370; Bayer Schering Pharma, Osaka, Japan, or Omnipaque 350; GE healthcare Japan, Tokyo). The bolus tracking technique was used to determine the timing of the dynamic study; arterial phases were obtained at 5.3 s after the 100-HU elevation of CT attenuation in the descending aorta. The portal venous and delayed phases were acquired at 40 s and 240 s after the initiation of contrast material injection, respectively. The imaging acquisition parameters were as follows: rotation time 0.5 s, 64 × 0.625 mm collimator, tube voltage 120 kV, automated tube current modulation, field of view 32 cm, image reconstruction thickness 1 mm, and spiral pitch 0.797.

### Imaging analysis

The spectral-based image data of the three phases were postprocessed with a dedicated workstation (IntelliSpace Portal; Philips Electronics Japan). Two radiologists (D.T. and N.T.) identified the lesions on conventional 120 keV CT images in consensus using endoscopy records as references. They also in consensus evaluated qualitative CT and quantitative dlDECT parameters. The quantitative CT parameters included contour (round or lobulated), growth pattern (endoluminal or exophytic), enhancement pattern (homogeneous, heterogeneous), and presence or absence of necrosis. As a quantitative dlDECT analysis, the readers placed the largest-possible circular region of interest (ROI) that avoided vessels, calcifications, or cystic lesions. The distribution of ROI included three in cardia, four in fundus, and 19 in body. Another ROI was placed in the aorta of the same slice in each case for preparing normalization. The mean iodine density (mg/ml) of the lesion and aorta was calculated, and physiological variations in patients could be minimized using the normalized iodine concentration (NIC) = IC_lesion_/IC_aorta_. The spectral slope (*λ*_HU)_ was adopted as a second dlDECT parameter. The spectral slope reflects the variation of the material CT value with the energy of the X-ray and absorption characteristics relative to different X-ray energies [12]. We calculated the slope using the values of 40 keV and 70 keV monochromatic images according to the following formula: *λ*_HU_ = (CT40 keV–CT70 keV)/30. These measurement processes were performed for all three phases.

### Statistical analysis

Continuous and categorical variables were examined by Wilcoxon rank sum test and the Chi-square test or Fisher’s exact test. We compared the clinical features of patients and dlDECT parameters (NIC and *λ*_HU_) of GISTs and leiomyomas. *P* values less than 0.05 were considered to indicate statistical significance. Receiver-operating characteristic (ROC) curves were plotted and the areas under the curve (AUCs) were calculated to determine the diagnostic efficiency using parameters from the tests above that had shown significance in differentiating between GISTs and leiomyomas. We determined optimal cut-off value according to the ROC curves mathematically using Youden’s index, and the corresponding sensitivities and specificities were also extracted. Statistical analysis was performed using SPSS 18 for Windows software (SPSS, Chicago, IL, USA).

## Results

**Fig. 1:**
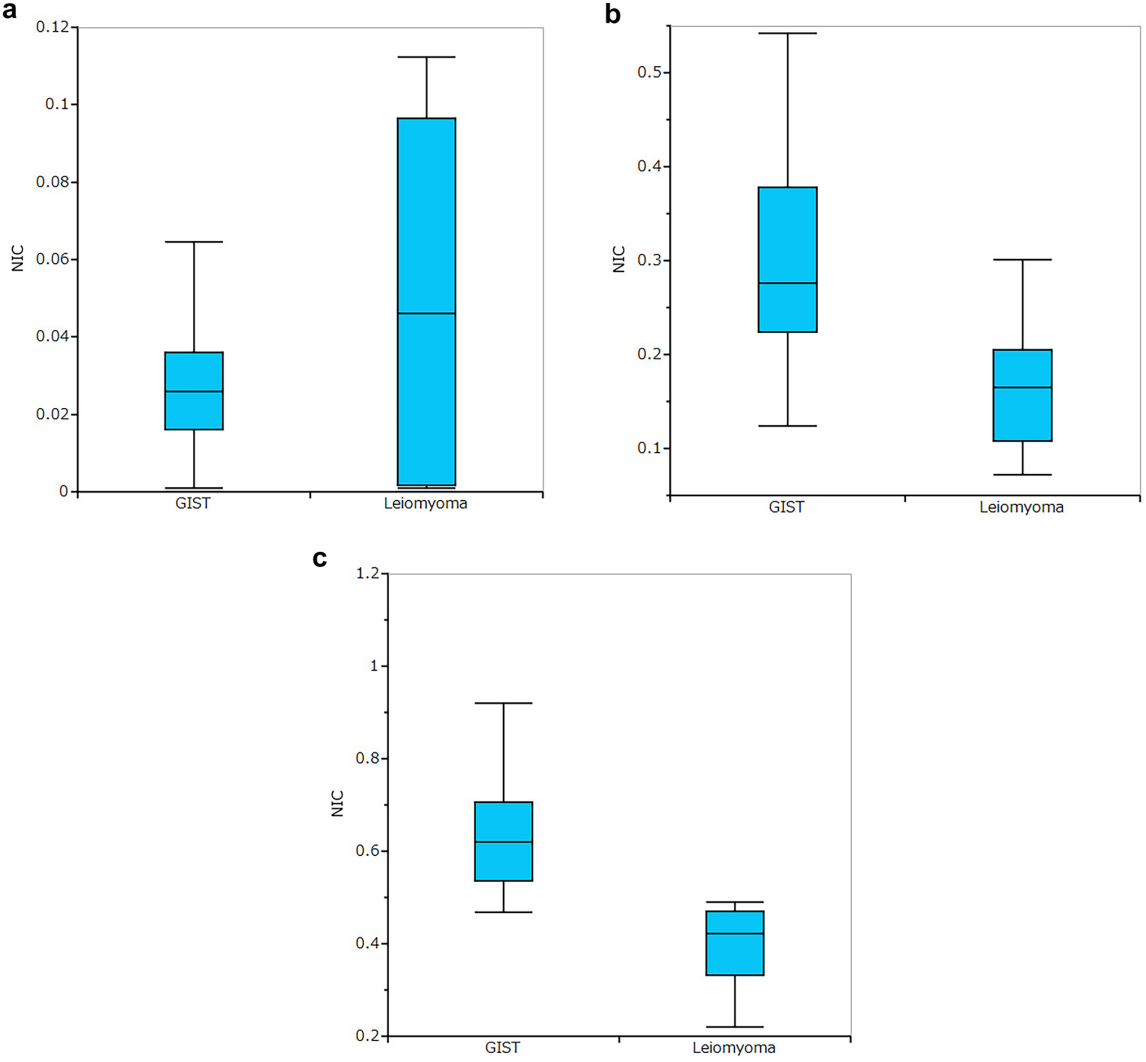
Box plot showing the range of NIC for GISTs and leiomyomas at the **A** arterial, **B** portal, and **C** delayed phases. The NIC was significantly higher for GIST lesions than leiomyomas on the portal and delayed phases

**Fig. 2:**
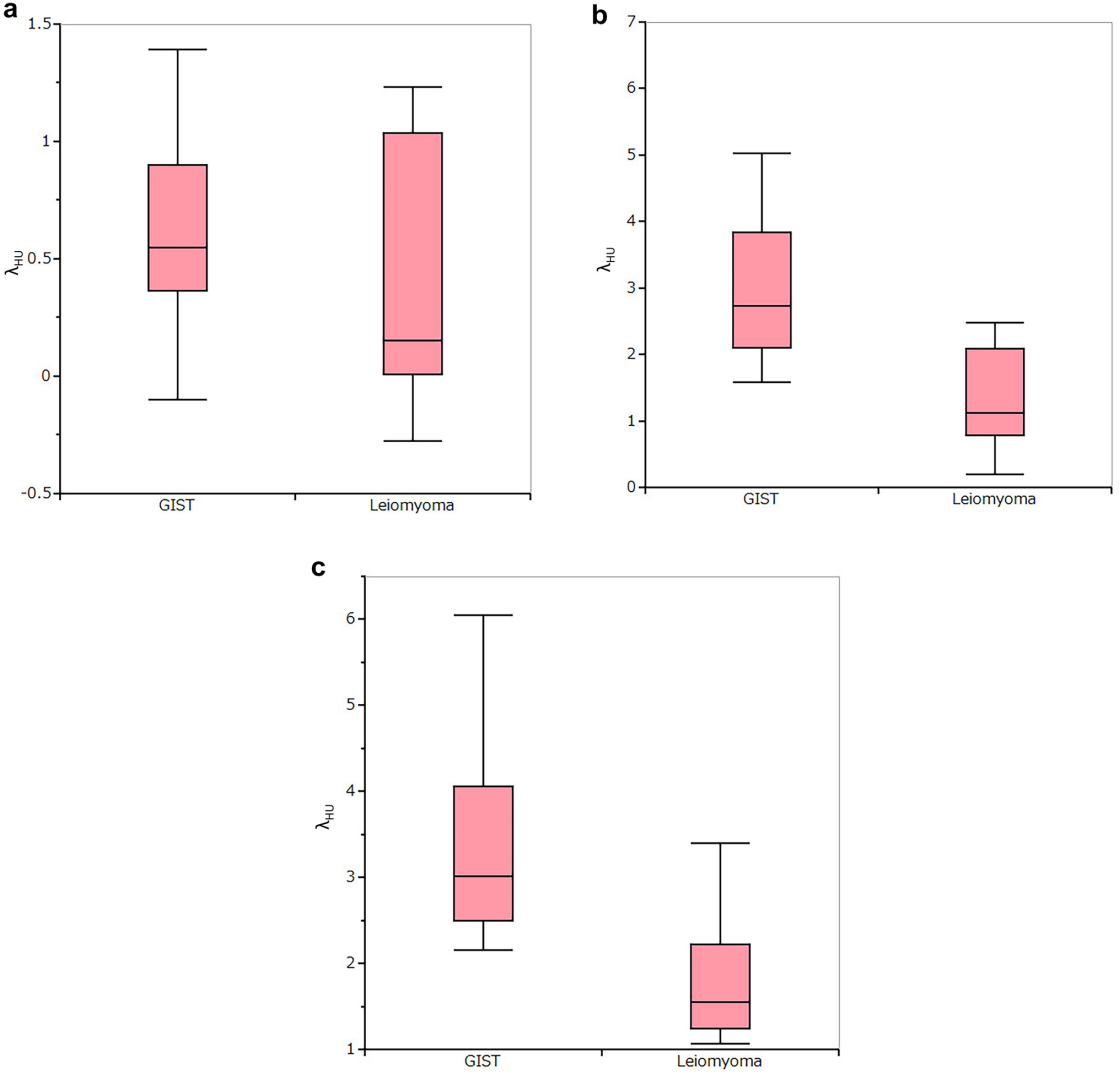
Box plot showing the range of *λ*_HU_ for GISTs and leiomyomas at the **A** arterial, **B** portal, and **C** delayed phases. *λ*_HU_ was significantly higher in GIST lesions than leiomyomas on the portal and delayed phases

The qualitative CT parameters were not significantly different between GISTs and leiomyomas (Table 2). The dlDECT parameters of GISTs and leiomyomas are summarized in Table 3. NIC was significantly higher in GISTs than in leiomyomas in both the portal (*P* = 0.0019) and delayed phases (*P* = 0.0011). There was no statistically significant difference in the arterial phase (*P* = 0.5216) (Fig. 1). *λ*_HU_ was significantly higher in GISTs than in leiomyomas in the portal (*P* = 0.0006) and delayed phases (*P* = 0.0009). There was no statistically significant difference at the arterial phase (*P* = 0.1866) (Fig. 2).

**Table 2:** Qualitative CT parameters of gastric GIST and leiomyoma

	GIST (*n* = 17)	Leiomyoma (*n* = 9)	*P*
Contour			
Round	16	8	0.634
Lobulated	1	1	
Growth pattern			
Endoluminal	14	8	0.660
Exophytic	3	1	
Enhancement			
Homogeneous	17	9	NA
Heterogeneous	0	0	
Necrosis			
Present	0	0	NA
Absent	17	9	

*NA* not applicable

**Table 3:** dlDECT parameters of gastric GIST and leiomyoma

	GIST (*n* = 17)	Leiomyoma (*n* = 9)	*P*
*λ*_HU_, median (range)			
AP	0.025 (0.001–0.093)	0.046 (0.001–0.112)	0.5216
PP	0.276 (0.124–0.542)	0.165 (0.072–0.301)	0.0019
DP	0.619 (0.467–1.148)	0.421 (0.221–0.767)	0.0011
NIC, median (range)			
AP	0.546 (− 0.443–1.393)	0.153 (− 0.276–1.230)	0.1866
PP	2.726 (1.576–6.753)	1.120 (0.190–2.476)	0.0006
DP	3.016 (2.150–6.050)	1.546 (1.063–3.400)	0.0009

*NIC* normalized iodine concentration, *AP* arterial phase, *PP* portal phase, *DP* delayed phase

**Fig. 3:**
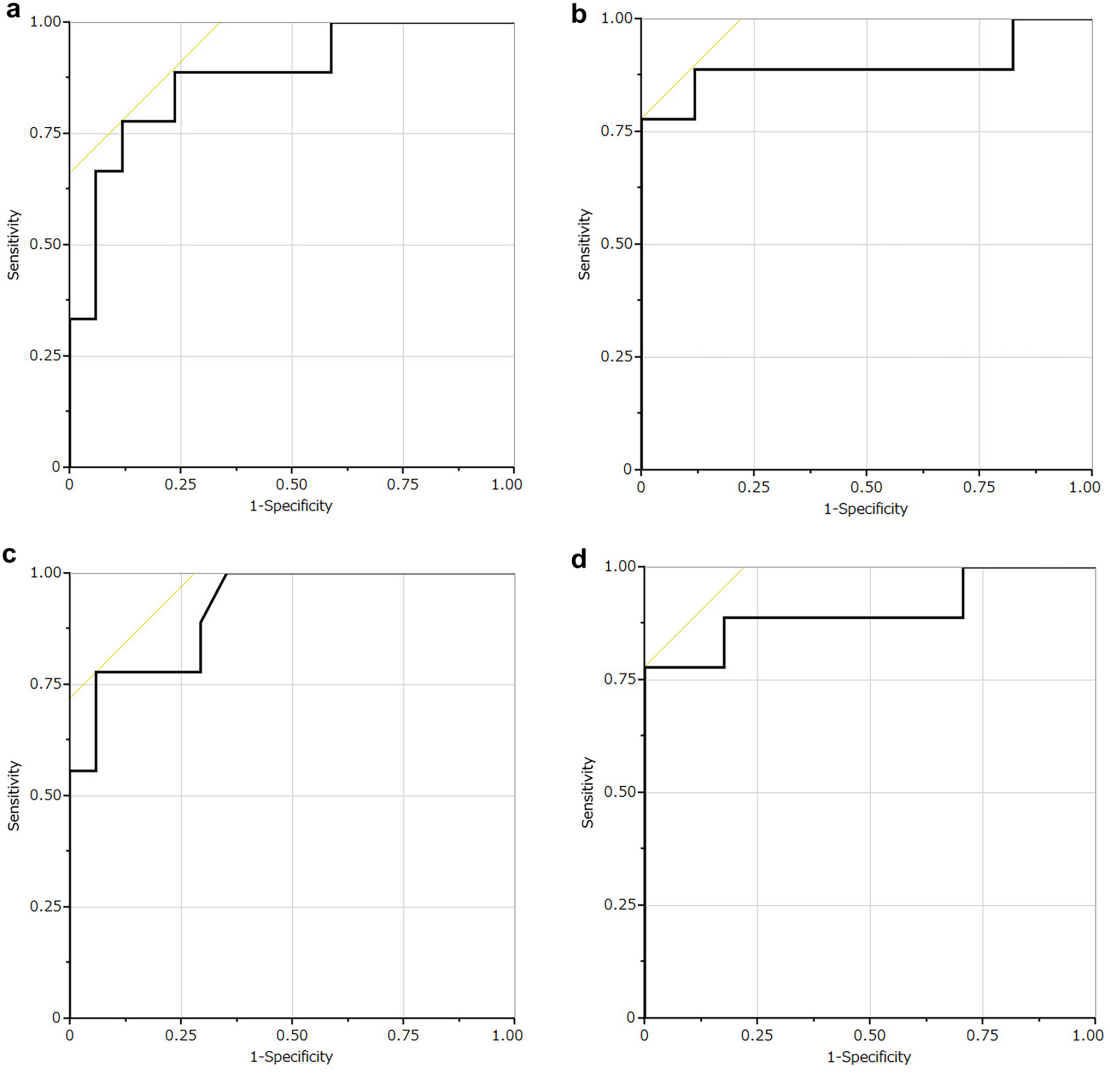
ROC curve analyses of NIC (**A** portal phase; **B** delayed phase) and *λ*_HU_ (**C** portal phase; **D** delayed phase) for distinguishing GISTs from leiomyomas

**Fig. 4:**
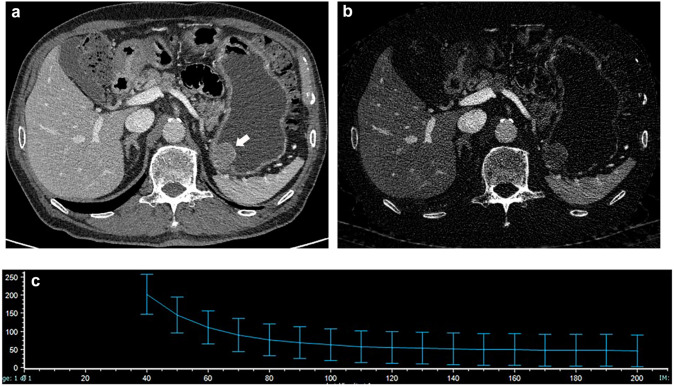
An 81-year-old man with a GIST. **A** CT image at the portal phase shows a well-defined round mass in the gastric fundus (arrow). **B** NIC of the lesion was 0.33 on the iodine-based image. **C** The spectral curve of the portal phase. *λ*_HU_ was calculated to be 3.85

**Fig. 5:**
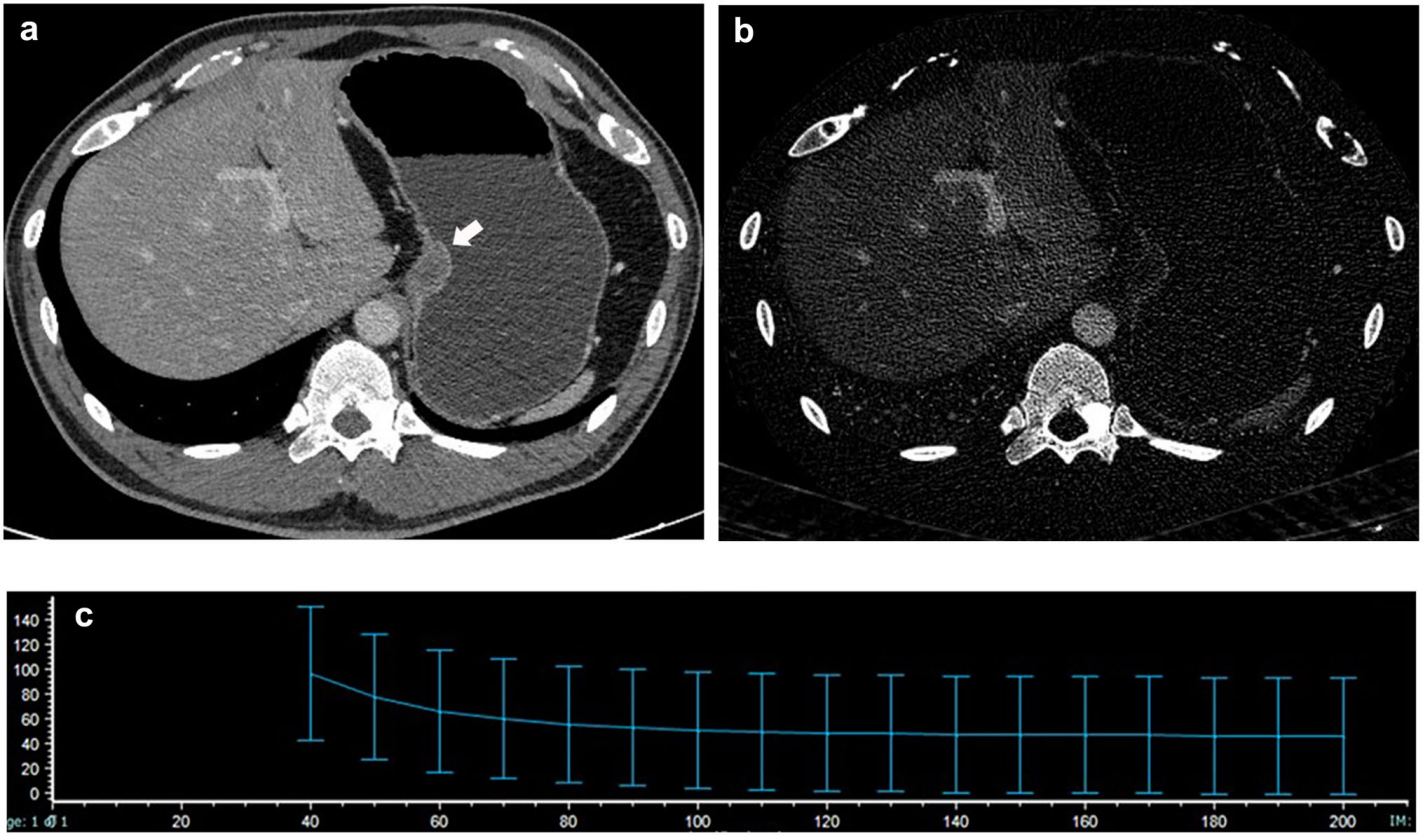
A 46-year-old man with a leiomyoma. **A** CT image at the portal phase shows a well-defined oval mass in the gastric cardia (arrow). **B** The NIC of the lesion was 0.12 on the iodine-based image. **C** The spectral curve of the portal phase. *λ*_HU_ was calculated to be 1.11

In the ROC analyses, the AUCs of the ROC curves using NIC to differentiate between GISTs and leiomyomas were 0.875 and 0.895 in the portal and delayed phase; using *λ*_HU_, they were 0.918 and 0.902, respectively (Fig. 3). When the cut-off value for NIC was set at 0.222 and 0.489, the diagnostic performance showed sensitivities of 88.9% and 88.9% and specificities of 76.5% and 88.2% in the portal and delayed phase, respectively. When the cut-off value for *λ*_HU_ was set at 1.720 and 2.123, the diagnostic performance showed sensitivities of 77.8% and 77.8% and specificities of 94.1% and 100.0% in the portal and delayed phase. Examples of the images of GIST and leiomyoma are shown in Figs. 4 and 5.

## Discussion

The results of our study showed that the values of NIC and *λ*_HU_ of GIST were significantly higher than those of leiomyoma in both the portal and delayed phase. Several prior studies have presented methods of differential diagnosis on CT between gastric GIST and leiomyoma, which manifest similarly as common gastric SMTs with overlapping symptoms and gross radiological features [3, 7, 13]. Choi et al. attempted to explore CT findings to differentiate GIST from non-GIST using cohort of large (≥ 5 cm) gastric SMTs. Their results showed that non-cardial location, heterogeneous enhancement, and presence of necrosis were differential CT features indicating GIST rather than leiomyoma [3]. Zhu et al. analyzed a cohort of 41 gastric SMTs using biphasic contrast-enhanced CT to differentiate gastric leiomyoma from GIST. They found that cardial location, round/ovoid contour, intraluminal growth, homogeneous enhancement, absence of necrosis, less than 3.35 cm in long diameter and less than 2.3 cm in short diameter, and enhancement degree of less than 12.5 HU in the arterial phase versus less than 31.5 HU in the portal phase were significant features of true gastric leiomyoma [7]. Recently, Xu et al. built a diagnostic scoring model using multiple CT features for differentiating gastric leiomyoma from GIST. The combined CT features, including esophagogastric junction involvement, absence of ulceration and necrosis, mild enhancement, and high long-diameter/short-diameter ratio, achieved high diagnostic performance with a sensitivity of 95.6% and specificity of 79.4% [13]. Accordingly, the large size including a greater long diameter has been a key feature in diagnosing GISTs. Conversely, it is challenging to diagnose smaller GIST by the conventional CT image findings as shown in the qualitative CT analysis of this study. Some previous reports analyzing the size criteria have defined submucosal tumors < 5 cm in diameter as “small submucosal tumors”; among the tumors of their study cohort, GISTs tended to show a homogenous mass with regular shape indistinguishable from benign tumors [8, 9]. Kim et al. attempted to analyze CT features of gastric SMTs limited to the size of 4 cm or smaller focusing on differentiation from ectopic pancreas. They treated GIST and leiomyoma together as a differential diagnosis of ectopic pancreas, suggesting that the imaging features of the two entities are quite similar [4]. It has been widely accepted that discrimination of small gastric GIST lesions from other SMTs including leiomyoma is very difficult even with the use of EUS, which has a diagnostic accuracy ranging from 45.5 to 48.0% [14–16]. Therefore, invasive diagnostic procedures, such as EUS-FNAB, are recommended, depending on applicable guidelines or local rules [17, 18]. The results of the present study enrolling a cohort of small gastric SMTs, with a median diameter of 14.5 mm, showed the high diagnostic performance using dlDECT parameters, with sensitivity ranging from 77.8 to 88.9% and specificity ranging from 76.5 to 100.0%. The results would be better than reported results using conventional contrast-enhanced CT showing the sensitivity and specificity of 82% and 77% in differentiating GISTs and leiomyomas even for larger tumors [7]. These findings provide the necessary diagnostic confidence before attempting an invasive procedure. Histopathologically, leiomyomas are low-to-moderately cellular and vascularity tumors, whereas GISTs show a higher overall cellularity and vascularity [19]. These histological differences may have influence on image features in dlDECT. Liu et al. compared the Spectral CT findings of GIST and schwannoma of the stomach using the arterial and portal phase. According to the results, GIST showed significantly higher iodine concentration and slope of the spectral curve on arterial and portal phase than schwannoma. They speculated that the tissue difference between GIST and schwannoma may be specifically depicted on spectral CT [11]. Zhang et al. examined the value of Spectral CT quantitative parameters in GIST risk classification using arterial, portal, and delayed phase, as in our study. According to their results, GIST with high risk showed significantly higher NIC than that with intermediate and low risk on each of the enhanced phases. They proposed one potential explanation for these results: namely, that a high-risk GIST is more vascular with larger and increased numbers of supplying blood vessels, thereby increasing the endothelial gap and resulting in an increase in the permeability of the tumor. The slope of the spectral curve of high-risk GIST was also significantly higher than that of intermediate- and low-risk GIST on each of the enhanced phases. The differences in the spectral curve derive from the differences in the material CT value of chemical molecular structure depending on the energy of the X-ray and absorption characteristics [10]. A similar mechanism may underlie our present findings that both NIC and λ_HU_ of GIST lesions were significantly higher than those of leiomyomas.

There are several limitations of this study. First, it was a single-institution study with a small sample size, which probably due to clinical background described as the second limitation below. Second, the study cohort included patients with gastric SMTs and no other specific findings who were invited to our institution for the purpose of extensive examination (for small-sized lesions), which may have introduced selection bias. Third, the imaging analyses performed by two radiologists in consensus may not have been fully objective or reproducible. However, gastric SMTs, even small tumors, tend to manifest as round- or oval-shaped mural nodules, which would not cause a great measurement bias.

In conclusion, dlDECT parameters, including NIC and *λ*_HU_, show promise as indicators to differentiate GISTs from leiomyomas, specifically for small-sized (≤ 4 cm) gastric SMTs, to gain the desired diagnostic confidence before undertaking an invasive procedure to obtain histological specimens.
